# Characteristics of tinnitus in workers exposed to occupational noise: a systematic review

**DOI:** 10.47626/1679-4435-2025-1466

**Published:** 2025-09-22

**Authors:** Eduarda Gabrielle Azambuja Freire, Taís de Azevedo Picinini, Adriana Laybauer Silveira Unchalo, Adriane Ribeiro Teixeira

**Affiliations:** 1 Curso de Fonoaudiologia, Universidade Federal do Rio Grande do Sul (UFRGS), Porto Alegre, RS, Brazil; 2 Programa de Pós-Graduação (Doutorado) em Distúrbios da Comunicação, Universidade Tuiuti do Paraná, Curitiba, PR, Brazil; 3 Serviço de Fonoaudiologia, Hospital de Clínicas de Porto Alegre (HCPA), Porto Alegre, RS, Brazil; 4 Departamento de Saúde e Comunicação Humana, UFRGS, Porto Alegre, RS, Brazil

**Keywords:** tinnitus, noise, occupational, noise., zumbido, ruído ocupacional, ruído.

## Abstract

Tinnitus is an auditory symptom characterized by the perception of sound without an external source and is often associated with occupational noise exposure. This study aimed to analyze the possible characteristics of tinnitus in workers exposed to occupational noise. This is a systematic literature review conducted by researchers who independently performed the search and analysis of articles. Searches were conducted in the PubMed, Embase, Latin American and Caribbean Health Sciences Literature (LILACS), Scopus, and Web of Science databases as well as Google Scholar, used as a source of gray literature. The review question was: “What are the characteristics of tinnitus in workers exposed to occupational noise?” Descriptors were selected from the Medical Subject Headings and the Health Sciences Descriptors. A total of 1,002 studies were identified, and after analysis, only 7 were included in the review. Studies indicated that tinnitus was predominantly perceived in both ears, with a high-pitched tone. Loudness was classified as mild to moderate. The type of tinnitus was described as a “whistle” or “hissing.” Periodicity was mostly intermittent. In individuals exposed to occupational noise, tinnitus is bilateral, high-pitched, mild to moderate in loudness, tonal, and intermittent. Monitoring tinnitus and its characteristics in workers is essential for auditory and mental health.

## Abstract (pt)

O zumbido é um sintoma auditivo caracterizado pela percepção de sons sem uma fonte externa, sendo frequentemente relacionado à exposição ao ruído ocupacional. Este estudo teve como objetivo analisar as possíveis características do zumbido em trabalhadores expostos ao ruído ocupacional. Trata-se de uma revisão sistemática da literatura, conduzida por pesquisadoras que, de forma independente, realizaram a busca e a análise dos artigos. As buscas foram realizadas nas bases de dados PubMed, Embase, Literatura Latino-Americana e do Caribe em Ciências da Saúde (LILACS), Scopus, Web of Science, além do Google Scholar, utilizado como fonte de literatura cinzenta. A pergunta de pesquisa foi: “Quais são as características do zumbido em trabalhadores expostos ao ruído ocupacional?”. Os descritores utilizados foram selecionados a partir do Medical Subject Headings e dos Descritores em Ciências da Saúde. Foram identificados 1.002 estudos e, após análise, apenas sete foram incluídos no trabalho. Os estudos indicaram que o zumbido foi predominantemente percebido nas duas orelhas, com *pitch* descrito como alto. O *loudness* foi classificado como de leve a moderado. O tipo de zumbido foi descrito como “apito” e “assobio/sibilante”. A periodicidade foi majoritariamente intermitente. Conclui-se que, em indivíduos expostos ao ruído ocupacional, o zumbido é bilateral, de *pitch* alto, *loudness* de leve a moderado, tonal e intermitente. Monitorar o zumbido e suas características em trabalhadores é essencial para a saúde auditiva e mental.

## INTRODUCTION

Many occupations expose workers to environments with high and prolonged noise levels, which can cause health damage.^1^ In Brazil, Regulatory Standard No. 15, established by Ordinance No. 3,214/1978, defines the exposure limits for continuous, intermittent, and impact noise, allowing a maximum of 8 hours per day for noise levels of 85 decibel (dB[A]).^2^

Among the auditory effects caused by noise, hearing loss is widely recognized. Other disorders may also occur, such as tinnitus and hyperacusis, all initially triggered by damage to the outer hair cells. Continued exposure can, over time, lead to changes in the inner hair cells and stria vascularis.^3^

Tinnitus can be described as an auditory symptom characterized by the perception of sound without an external source. The term *tinnitus* comes from the Latin verb *tinnire*, meaning “to ring.”^4^ Epidemiological studies worldwide report that the prevalence of tinnitus ranges from 5% to 43%, increasing with age.^5^

One study indicates that tinnitus rates are significantly higher among individuals who work continuously in noisy environments, suggesting that both the duration and intensity of exposure influence the development of the symptom.^6^ Thus, even with mandatory hearing protection and compliance with current legislation, the prevalence of tinnitus among workers exposed to occupational noise remains high.^7^

Although the literature reports the presence of tinnitus in workers exposed to occupational noise, there is still no clear definition of its characteristics in this population. These characteristics can be analyzed through interviews and specific assessments, such as acuphenometry, in which the audiologist performs tests to identify the pitch (perceived frequency) and loudness (perceived intensity) experienced by the individual in relation to the symptom.

Because of the high number of workers exposed to noisy environments who report auditory changes such as tinnitus, conducting this study is particularly relevant. A deeper understanding of the characteristics of the symptom in this population will provide further insight into the condition. Comprehending and detailing these aspects is essential for identifying effective prevention and management strategies, contributing to greater safety in the workplace. Therefore, the current study aims to examine the possible characteristics of tinnitus in workers exposed to occupational noise.

## METHODS

### SEARCH STRATEGY

This study is a systematic review conducted as per the Preferred Reporting Items for Systematic Reviews and Meta-Analyses (PRISMA) recommendations and followed the steps established by the Cochrane Handbook for Systematic Reviews of Interventions (Cochrane, 2023), namely: formulating the review question; locating and selecting studies; critically appraising studies; collecting data; analyzing and presenting data; interpreting results; and improving and updating the review.^8,9^

Initially, a search was performed in the PROSPERO database to verify whether other systematic reviews on this topic existed. As none were found, the review was registered (CRD42025649367) and was continued. The study was also registered on the Open Science Framework platform (DOI 10.17605/OSF.IO/Z5WJQ).

The review question was developed using the PECOS acronym, where P (population) refers to individuals with tinnitus; E (exposure) refers to occupational noise exposure; C (comparison) refers to comparing tinnitus among noise-exposed individuals; O (outcome) refers to the characteristics of tinnitus as described and assessed by professionals; and S (study design) refers to the study designs included (cross-sectional).

Accordingly, the review question was defined as: “What are the characteristics of tinnitus in workers exposed to occupational noise?”

Studies were searched in the PubMed, Embase, Scopus, Latin American and Caribbean Health Sciences Literature (LILACS), and Web of Science databases as well as Google Scholar, which was used as a source of gray literature. Access to the databases was obtained through the CAFe portal, available from periodicos.capes.gov.br.

The selected descriptors were identified from the Health Sciences Descriptors and the Medical Subject Headings, namely: tinnitus OR noise induced tinnitus OR pulsatile tinnitus OR ear buzzing OR tinnitus aurium OR clicking tinnitus OR objective tinnitus OR subjective tinnitus OR spontaneous oto-acoustic emission tinnitus OR spontaneous oto acoustic emission tinnitus AND noise, occupational OR occupational noise OR noises, occupational OR occupational Noises AND noise OR noises OR noise pollution (Table 1).

**Table 1:** Search strategies for each database

Database	Search strategy
PubMed	(“tinnitus” [Mesh Terms] OR “tinnitus” OR “noise induced tinnitus” OR “pulsatile tinnitus” OR “ear buzzing” OR “tinnitus aurium” OR “clicking tinnitus” OR “objective tinnitus” OR “subjective tinnitus” OR “spontaneous oto-acoustic emission tinnitus” OR “spontaneous oto acoustic emission tinnitus”) AND (“noise, occupational” [Mesh Terms] OR “noise, occupational” OR “occupational noise” OR “noises, occupational” OR “occupational noises”) AND (“noise” [Mesh Terms] OR “noise” OR “noises” OR “noise pollution”)
Embase	(‘tinnitus’ OR ‘noise induced tinnitus’ OR ‘pulsatile tinnitus’ OR ‘ear buzzing’ OR ‘tinnitus aurium’ OR ‘clicking tinnitus’ OR ‘objective tinnitus’ OR ‘subjective tinnitus’ OR ‘spontaneous oto-acoustic emission tinnitus’ OR ‘spontaneous oto acoustic emission tinnitus’) AND (‘noise, occupational’ OR ‘occupational noise’ OR ‘noises, occupational’ OR ‘occupational noises’) AND (‘noise’ OR ‘noises’ OR ‘noise pollution’)
LILACS	(“tinnitus” OR “acúfeno” OR “zumbido”) AND (“noise, occupational” OR “ruido en el ambiente de trabajo” OR “ruído ocupacional”) AND (“noise” OR “ruido” OR “ruído”)
Web of Science	(“tinnitus” OR “noise induced tinnitus” OR “pulsatile tinnitus” OR “ear buzzing” OR “tinnitus aurium” OR “clicking tinnitus” OR “objective tinnitus” OR “subjective tinnitus” OR “spontaneous oto-acoustic emission tinnitus” OR “spontaneous oto acoustic emission tinnitus”) AND (“noise, occupational” OR “occupational noise” OR “noises, occupational” OR “occupational noises”) AND (“noise” OR “noises” OR “noise pollution”)
Scopus	(“tinnitus” OR “noise induced tinnitus” OR “pulsatile tinnitus” OR “ear buzzing” OR “tinnitus aurium” OR “clicking tinnitus” OR “objective tinnitus” OR “subjective tinnitus” OR “spontaneous oto-acoustic emission tinnitus” OR “spontaneous oto acoustic emission tinnitus”) AND (“noise, occupational” OR “occupational noise” OR “noises, occupational” OR “occupational noises”) AND (“noise” OR “noises” OR “noise pollution”)
Google Scholar	“tinnitus” OR “noise induced tinnitus” OR “pulsatile tinnitus” OR “ear buzzing” OR “tinnitus aurium” OR “clicking tinnitus” OR “objective tinnitus” OR “subjective tinnitus” OR “spontaneous oto-acoustic emission tinnitus” OR “spontaneous oto acoustic emission tinnitus” AND “noise, occupational” OR “occupational noise” OR “noises, occupational” OR “occupational noises” AND “noise” OR “noises” OR “noise pollution” filetype

LILACS = Latin American and Caribbean Literature on Health Sciences.

### SELECTION CRITERIA

For inclusion, studies were required to involve workers exposed to occupational noise who presented with tinnitus and to provide information on the characteristics of the reported symptom. Studies in Portuguese, English, Spanish, and French were considered, with no restriction on publication year.

Exclusion criteria comprised materials such as reviews, letters to the editor, editorials, and opinion articles, which do not provide original data. In addition, studies involving nonoccupational noise or animal models were excluded.

Study selection and review were performed independently by 2 review authors. The selection process included the following steps: removal of duplicate titles, screening of titles and abstracts, and full-text reading. In cases of disagreement, a third review author (arbiter) was consulted to make the final decision.

### DATA ANALYSIS

Searches were conducted independently by 2 review authors, and the retrieved studies were imported into the Zotero reference manager. Records were then exported to the Rayyan platform, where study screening was conducted. The process began with the removal of duplicates, followed by the screening of titles and abstracts of the remaining studies. Those meeting the inclusion criteria underwent full-text reading. After this stage, the studies to be included in the review were selected, based on the predefined inclusion and exclusion criteria. In cases of disagreement between the 2 review authors, a third one was called in as an arbiter to make the final decision on the inclusion or exclusion of the disputed articles.

The methodological quality of the studies was assessed using the Grading of Recommendations, Assessment, Development and Evaluation (GRADE) system, developed to classify the quality of evidence and the strength of recommendations. This system provides a reliable basis for health care decision-making by assessing the level of confidence in effect estimates.^10^

GRADE classifies evidence into 4 levels (high, moderate, low, and very low), considering various aspects such as study design and execution, consistency of results, possible limitations, indirect evidence, missing data, and risk of bias.^10^

## RESULTS

**Figure 1:**
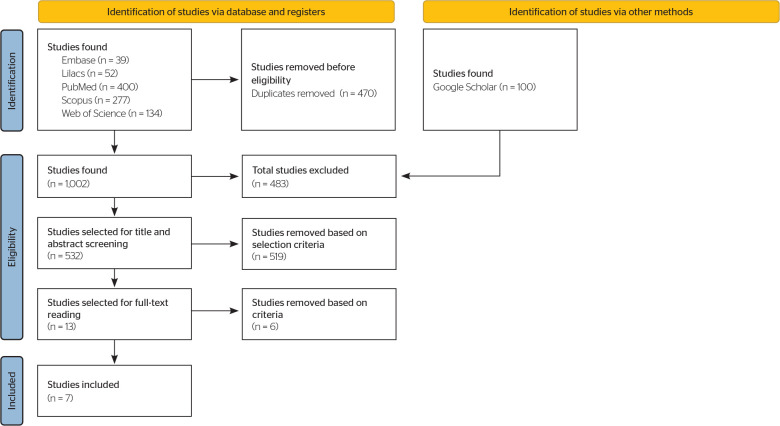
Flowchart of the literature search and selection criteria adapted from the Preferred Reporting Items for Systematic Reviews and Meta-Analyses (PRISMA).

A total of 1,002 studies were identified, of which 470 duplicates were removed, leaving 532 for title and abstract screening. Of these, 519 were excluded for not meeting the inclusion criteria, resulting in 13 studies selected for full-text reading. During this stage, 8 studies showed disagreements between the review authors, which led to the involvement of a third one who, after full reading, excluded 6 studies that did not provide information on characteristics of tinnitus or were review articles. At the end of the process, 7 studies were included in the systematic review.^11-17^ The search and selection process is shown in a flowchart (Figure 1). Based on this process, 7 articles were selected, and information such as author and publication year, objective, sample, and tinnitus characteristics in workers exposed to occupational noise is presented (Table 2).

**Table 2:** Description of study characteristics

Authors and year of publication	Article title	Research objectives	Study sample and method of assessment of tinnitus	Characteristics of tinnitus
Phoon, Lee, Chia^11^/1993	Tinnitus in Noise-Exposed Workers	To study the prevalence and characteristics of tinnitus in a group of noise-exposed workers reported to the Ministry of Labor as cases of noise-induced hearing loss.	Workers exposed to occupational noise (metal industry), totaling 647 participants. Tinnitus investigated through interviews.	A total of 66% of participants reported tinnitus, with 41.5% experiencing it frequently, either bilaterally (41.5%) or unilaterally (30.2% in the left ear and 28.3% in the right ear). Tinnitus was described as a “whistle,” leading researchers to conclude it was high-frequency.
Jansen, Helleman, Dreschler, Laat^12^/2009	Noise Induced Hearing Loss and Other Hearing Complaints Among Musicians of Symphony Orchestras	To investigate hearing aspects of professional symphony orchestra musicians.	Musicians from 5 symphony orchestras, totaling 241 participants and 482 ears analyzed. Acuphenometry was performed for tinnitus analysis.	Tinnitus was present in 51% of participants. Most reported unilateral tinnitus, described as high-frequency, with frequencies above 4,000 Hz.
Dejonckere, Coryn, Jean Lebacq^13^/2009	Experience with a Medicolegal Decision-Making System for Occupational Hearing Loss-Related Tinnitus	To investigate the audiological characteristics linking chronic tinnitus to NIHL.	Patients claiming compensation for occupational hearing loss and tinnitus. Medical records were analyzed and acuphenometry data reviewed.	In 31 analyzed cases, tinnitus was bilateral, and in 4, unilateral (predominantly in the right ear), with pitch at 4,000 Hz and loudness of 7.20 ± 3.4 dB SL.
Steinmetz, Zeigelboim, Lacerda, Morata, Marques^14^/2009	The characteristics of tinnitus in workers exposed to noise	To study tinnitus characteristics in noise-exposed workers.	A total of 52 workers from a food industry. Tinnitus evaluated through interviews.	Predominantly bilateral, described as “hissing” and intermittent (weekly). Loudness described as “moderate.”
Weber, Périco^15^/2011	Zumbido no trabalhador exposto ao ruído [Tinnitus in workers exposed to noise]	To assess the characteristics, prevalence, and impact of tinnitus in noise-exposed workers.	Workers from a food industry company, totaling 42 participants. Tinnitus evaluated through interviews.	Bilateral tinnitus was reported in 50% of cases. Among unilateral cases, the symptom was more prevalent on the left side (21.4%). Most reported intermittent perception (88%) of a single sound (85.7%), with no description of the sound type provided by workers.
Flores, Teixeira, Rosito, Dall’Igna^16^/2016	Pitch and Loudness from Tinnitus in Individuals with Noise-Induced Hearing Loss	To analyze a possible association between gender and the frequency and intensity of tinnitus, as well as the degree of hearing loss and affected frequencies in individuals with NIHL.	Sample of 33 individuals, with tinnitus assessed through acuphenometry.	Unilateral tinnitus was reported by 61.7% of participants. Median pitch in both ears was 4,000 Hz, and median loudness was 15 dB SL in the right ear and 7.5 dB SL in the left ear.
Asghari^17^/2021	Tinnitus characteristics at high-and low-risk occupations from occupational noise exposure standpoint	To compare tinnitus characteristics, such as frequency, laterality, and associated hearing loss, between workers in occupations with different levels of noise exposure.	Participants divided into 2 groups (high risk and low risk) for tinnitus, based on the level of occupational noise exposure. Tinnitus was evaluated through interviews.	In the high-risk group, 6.84% had predominantly unilateral tinnitus on the left side. In the low-risk group, 5.6% had predominantly bilateral tinnitus.

dB SL = decibel sensation level; NIHL = noise-induced hearing loss.

LILACS = Latin American and Caribbean Literature on Health Sciences.

Tinnitus laterality was predominantly bilateral in 4 of 7 studies.^11,13-15^ In cases of unilateral tinnitus, the symptom was more frequently reported in the left ear.^12,16^

Regarding tinnitus pitch, it was mostly high; in studies where acuphenometry was performed, values of 4,000 Hz were found.^13,16^ Tinnitus loudness ranged from 7.0 to 15.0 dB sensation level (dB SL) in 1 study, while another reported a mean of 7.2 dB SL. Another study did not specify loudness values but described tinnitus as “moderate.”^14^

Some studies described the type of tinnitus as a “whistle,” “hissing,” or “buzzing sound.”^11,16^ As for periodicity, findings indicated it could be either frequent or intermittent.^11,15^

According to the GRADE system analysis, 4 studies were classified as having moderate-level evidence and 3 as having low-level evidence (Table 3).

**Table 3:** Level of scientific evidence of the studies analyzed according to the GRADE system

Title	Country/year	Method	Level of evidence
Tinnitus in Noise-Exposed Workers	Singapore/1993	Cross-sectional study	Moderate
Noise induced hearing loss and other hearing complaints among musicians of symphony orchestras	Netherlands/2009	Cross-sectional study	Moderate
Experience with a Medicolegal Decision-Making System for Occupational Hearing Loss-Related Tinnitus	Belgium/2009	Cross-sectional study	Low
Pitch and Loudness from Tinnitus in Individuals with Noise-Induced Hearing Loss	Brazil/2016	Cross-sectional study	Moderate
Tinnitus characteristics at high-and low-risk occupations from occupational noise exposure standpoint	Iran/2021	Cross-sectional study	Moderate
The Characteristics of Tinnitus in Workers Exposed to Noise	Brazil/2009	Cross-sectional study	Low
Zumbido no trabalhador exposto ao ruído [Tinnitus in workers exposed to noise]	Brazil/2011	Cross-sectional study	Low

GRADE = Grading of Recommendations, Assessment, Development and Evaluation.

## DISCUSSION

This systematic review analyzed and compiled the characteristics of tinnitus in individuals exposed to occupational noise. The study applied specific inclusion and exclusion criteria, resulting in the selection and analysis of 7 articles.

The number of studies and their publication periods are noteworthy. One was published in the 1990s, while the others were published after the 2000s, with the most recent in 2021. Considering that noise-induced hearing loss is a widely studied topic, it was expected that assessment of tinnitus would also have a significant number of publications, particularly in recent years, when considerable research has been devoted to the symptom, its causes, characteristics, and treatment. Because of the known auditory effects of noise, the authors anticipated obtaining data suitable for a meta-analysis, generating sufficient information for hearing care professionals to use in their daily practice. However, this was not possible.

This is another important point to note. Although different guidelines exist for the evaluation of patients with tinnitus,^18,19^ in the field of occupational audiology, the results of such assessments do not appear to be widely published. This is evident when only 2 articles^12,16^ reported acuphenometry data, while the others relied solely on results obtained through questions about tinnitus, with subjective and qualitative responses regarding its characteristics, such as “mild intensity,” “moderate intensity,” and “high frequency.” This reliance on subjective descriptions makes direct comparison of results more challenging.

In the 2 studies presenting acuphenometry data, tinnitus loudness in workers exposed to occupational noise ranged from 7 to 15 dB SL. Another study described loudness as moderate. However, this perception may be significantly influenced by stress resulting from occupational noise exposure.^20^ Constant noise in the workplace not only contributes to the onset of tinnitus but can also induce stress in these workers, which in turn may exacerbate the perceived loudness of the symptom, worsening its impact on their quality of life.^6,20^

Regarding pitch, the included studies indicated that tinnitus was high-frequency (4,000 Hz or more, or described as a “whistle”). In some of the analyzed articles, hearing loss was also reported among participants, with noise exposure identified as the causal factor, which initially affects these frequencies.^12,16^ In literature, other studies reinforce this association, showing that tinnitus pitch often coincides with the frequency range where hearing loss is most pronounced.^21,22^ This can be explained by the reduction of auditory stimuli in specific regions of the inner ear, which may trigger a compensatory mechanism in the central nervous system.^23^ This process involves increased neural activity in the brain areas related to hearing, as damaged cochlear hair cells repeatedly discharge, continuously stimulating auditory nerve fibers and generating the perception of tinnitus - often an early indicator of auditory changes.^21,22^

Tinnitus was predominantly bilateral, which can be explained by the nature of the condition, as it is characterized by otological damage in both ears.^12,13,16,24^ Thus, tinnitus may be interpreted as a potential early marker of hearing damage, since its occurrence often coincides with bilateral changes in the auditory system.^23^

As for tinnitus type, most studies described the perception as a “whistle.”^11^ “Hissing” tinnitus, also common in the general population with the symptom, was reported as predominant in 1 study.^14^

Tinnitus periodicity was most often described as intermittent in 3 studies, aligning with another study that also noted the frequent use of this term in the population analyzed.^11,14,6,25^ This finding was unexpected, as continuous tinnitus is more prevalent among individuals not exposed to occupational noise. The occurrence of intermittent tinnitus warrants further investigation, as it may be attributed, for instance, to environmental masking caused by the high-noise settings in which workers spend most of their day.

It is therefore hypothesized that, in many cases, individuals may not perceive tinnitus due to the elevated noise levels to which they are exposed for most of the day. Another possibility is that tinnitus manifests only during certain situations, such as when individuals experience other health problems (eg, stress, medical conditions, or anxiety). Nonetheless, the fact that tinnitus is intermittent - although it may be considered less impactful than continuous tinnitus - still results in significant consequences for the work and personal lives of these individuals. It is therefore essential that companies pay closer attention to tinnitus and become more familiar with its characteristics.

Considering all these aspects, tinnitus - beyond being a possible early indicator of noise-induced hearing loss - has significant impacts on workers’ safety, efficiency, and quality of life.^7^ Its presence may impair concentration and reduce the perception of warning signals or important alerts, thereby increasing the risk of workplace accidents.^26^

According to the GRADE system, the 7 studies analyzed presented evidence classified as moderate and low, due to methodological limitations such as the predominant use of cross-sectional designs, small sample sizes, subjective assessment methods, and the lack of adequate control for factors that could influence the results. These constraints hinder interpretation and reduce the reliability of some conclusions. Nevertheless, the analyzed studies highlight the need for more robust future research to strengthen the available evidence.

Analyses of tinnitus in workers are essential, as it should be noted that tinnitus is a stressor often associated with anxiety. Individuals exposed daily to occupational noise already experience a considerable physical and psychological burden, since, in many cases, noise is among the factors that can lead to health disorders. Therefore, it is essential that they receive guidance not only on preventing this symptom but also on treatment options, which should be individualized to promote physical and emotional well-being. Moreover, there is a need to monitor tinnitus and its characteristics in workers exposed to occupational noise as a way to protect auditory and mental health, promoting preventive and educational actions within companies.

Notably, many studies do not include acuphenometry data, but only descriptions of tinnitus reported by the workers evaluated. This limited the results, as such descriptions are influenced by various factors, including language, educational level, prior knowledge of the topic, emotions, symptom exacerbation, among others. Therefore, it would be important to perform specific tinnitus assessments in workers who report the symptom, considering that it is one of the most common auditory symptoms and may be associated with a significant decline in quality of life.

## CONCLUSIONS

This study identified the main characteristics of tinnitus in workers exposed to occupational noise as follows: predominantly bilateral laterality; high pitch; loudness ranging from mild to moderate (5 to 15 dB SL); and intermittent periodicity.
